# Cross-Sectional Associations Between Depressive and Anxiety Symptoms and Disordered Eating Behaviors by Sex in University Students

**DOI:** 10.3390/jcm14134611

**Published:** 2025-06-29

**Authors:** Ana Ballesta-Castillejos, Valentina Díaz-Goñi, Bruno Bizzozero-Peroni, Estela Jiménez-López, José Francisco López-Gil, Isabel Antonia Martínez-Ortega, Arthur E. Mesas, Miriam Garrido-Miguel

**Affiliations:** 1 Health and Social Research Center, Universidad de Castilla-La Mancha, 16002 Cuenca, Spain; ana.ballesta@uclm.es (A.B.-C.); bruno.bizzozero.peroni@ki.se (B.B.-P.); estela.jimenezlopez@uclm.es (E.J.-L.); isabela.martinez@uclm.es (I.A.M.-O.); arthur.emesas@uclm.es (A.E.M.); miriam.garrido@uclm.es (M.G.-M.); 2 Faculty of Nursing, Universidad de Castilla-La Mancha, 02006 Albacete, Spain; 3 Higher Institute of Physical Education, Universidad de la República, Rivera 40000, Uruguay; 4 Aging Research Center, Department of Neurobiology, Care Sciences and Society, Karolinska Institutet and Stockholm University, 17165 Stockholm, Sweden; 5 Center for Biomedical Research Network in Mental Health (CIBERSAM), Instituto de Salud Carlos III, 28029 Madrid, Spain; 6 School of Medicine, Universidad Espíritu Santo, Samborondón 092301, Ecuador; josefranciscolopezgil@gmail.com; 7 Vicerrectoría de Investigación y Postgrado, Universidad de Los Lagos, Osorno 5290000, Chile

**Keywords:** depression, anxiety, eating disorders, university students, cross-sectional study

## Abstract

Depressive and anxiety symptoms are associated with a greater likelihood of disordered eating behaviors (DEBs), but the role of sex in these associations is unclear. **Objectives**: The aim of this study was to analyze the associations between depression, anxiety, and DEBs in a sample of Spanish university students. **Methods**: Depression was assessed with the Beck Depression Inventory-II (BDI-II), anxiety with the Generalized Anxiety Disorder (GAD-7) instrument, and DEBs with the Sick, Control, One stone, Fat, Food (SCOFF) questionnaire. Statistical analyses included generalized linear regression models adjusted for sociodemographic, body composition, and lifestyle covariates as the main confounders. **Results:** Among the 453 students analyzed (71.5% female), the frequencies of mild-to-severe depression, mild-to-severe anxiety, and of DEBs were higher in the females (42.0%, 77.5%, and 32.7%, respectively) than in the males (24.0%, 52.7%, and 20.2%, respectively). The results of the adjusted GLMs were similar for both the sexes, indicating higher estimated marginal means of the SCOFF total score and greater odds of DEBs among those with mild-to-severe depression or anxiety than among those without these conditions. **Conclusions**: Depression and anxiety symptoms are cross-sectionally associated with DEBs in Spanish university students of both sexes. Future prospective studies are needed to examine the direction of these associations separately for females and males.

## 1. Introduction

According to the World Health Organization, one in eight people worldwide suffers from a mental disorder [1], with anxiety and depression being the most common [2]. Specifically, the prevalence rates of depression and anxiety disorders among college students are 33.6% and 39.0%, respectively [3].

Eating disorders (EDs) are complex psychiatric disorders characterized by abnormal eating and weight control behaviors that can lead to serious health consequences, including significant psychological and physical impairment [4], a lower quality of life, a higher risk of suicide attempts, and higher mortality than both the general population and those with other psychiatric disorders [5,6]. Thus, EDs accounted for 17,361.5 years of life lost (between 1990 and 2019) and caused 318.3 deaths worldwide in 2019 [7]. Notably, the lifetime prevalence of EDs varies significantly by sex, being 4.9% in females and 2.9% in males [8].

It is important to distinguish between EDs, which are clinically diagnosed with psychiatric conditions, and disordered eating behaviors (DEBs), which refer to a range of irregular eating practices, such as restrictive dieting, binge eating, self-induced vomiting, and the misuse of laxatives or diuretics, that do not necessarily meet the diagnostic criteria for an ED [4,9]. However, although its impact on health is often neglected, DEBs need to be carefully assessed, as they can often develop into EDs [10,11].

Although EDs are distinct from mood disorders [12], their co-occurrence may be related to shared risk factors, such as genetic and environmental vulnerabilities [13]. Research has shown that people with depression, anxiety, and EDs tend to have highly negative emotionality, a personality trait associated with intense and frequent negative emotions [14,15,16]. In addition, having depression, anxiety, or an ED may increase the risk of developing another one of these disorders. For example, irregular eating patterns may negatively affect mood, and dysregulated eating may arise as a coping mechanism to regulate negative emotions [5]. Furthermore, elevated levels of anxiety, depression, or stress have been found to be associated with the use of dysfunctional emotion regulation strategies, such as binge eating, which are, in turn, linked to a greater eating disorder psychopathology. These factors appear to be mutually reinforcing, indicating a bidirectional relationship between emotional distress and DEBs [17,18].

Traditionally, EDs have been associated with the female population, and this stereotype has led men to face challenges such as stigmatization and delays in seeking help, as there is a perception that EDs are “women’s conditions” [19,20]. However, these disorders also pose a significant health risk to men, and although the prevalence of EDs in men is still lower than that in women, the number of cases has been steadily increasing in both sexes [6,21]. Furthermore, the symptoms tend to differ between men and women, as men with EDs are less concerned with thinness and more concerned with increasing body mass and muscularity, as well as having different patterns of emotional regulation [6,21,22].

There are various questionnaires and tools used to assess the symptoms of EDs, including the Eating Disorder Examination—Questionnaire (EDE-Q) [23]; the Eating Attitude Test (EAT-26) [24]; and the Sick, Control, One, Fat, Food (SCOFF) questionnaire [18]. The SCOFF questionnaire, developed in 1999 by Morgan et al. [18], is the most widely used tool to detect DEBs, which may lead to subsequent EDs.

Given that most cases of EDs (82.4%) develop in young adulthood [25] and that the consequences of these conditions are negative [5,6], particularly in women, our aim was to analyze whether the associations between depression, anxiety, and DEBs vary by sex among Spanish university students.

## 2. Materials and Methods

### 2.1. Study Design and Sample Size

This cross-sectional study was based on sociodemographic, anthropometric, lifestyle, and mental health data obtained from the “Nuts4Brain Project: the relationship between nut consumption and mental health outcomes throughout adulthood”. This study included students from the Universidad de Castilla-La Mancha (UCLM), Cuenca campus, Spain. The data were collected during the 2023–2024 academic year. The initial sample included 463 young adults (100.0%). However, in the present study, we analyzed the data from a subsample of 453 university students (97.8%), with complete data for all the study variables.

### 2.2. Ethics Approval and Consent to Participate

All the included university student participants met the following criteria: (i) aged between 18 and 30 years and (ii) without learning disabilities. The study protocol was validated and approved by the Clinical Research Ethics Committee of the “Hospital Virgen de la Luz”, in Cuenca, Spain (REG: 2023/PI1323) and complied with the principles of the Declaration of Helsinki. All the participants were informed of the conditions and characteristics of this study. Additionally, they were asked to sign an informed consent form as a requirement to participate in this study.

### 2.3. Dependent Variable: Disordered Eating Behaviors (DEBs)

The Spanish version of the validated SCOFF questionnaire was used to detect DEBs [26]. This self-administered instrument consists of five dichotomous questions (yes or no) that ask about vomiting, loss of control over overeating, body image distortion, weight loss, and the impact of food on life, as follows: (i) Do you make yourself vomit because you feel uncomfortably full? (ii) Are you concerned that you have lost control over how much you eat? (iii) Have you recently lost 1 stone (6 kg) in a 3-month period? (iv) Do you think you are fat when others say you are too thin? (v) Would you say that food dominates your life? The total score ranges from 0 to 5. A score ≥ 2 was used to indicate the presence of DEBs [27].

The SCOFF is a questionnaire that is applicable to both sexes, is simple and easy to administer and score, and has shown a sensitivity of 97.7% and a specificity of 94.4% for the screening of EDs in primary care [26,28].

### 2.4. Independent Variables: Depression and Anxiety

We used the Beck Depression Inventory-II (BDI-II) [29,30] to assess depressive state. This questionnaire has 21 multiple choice items about how the subject has experienced depression symptoms in the last week, reflecting the affective, cognitive, and somatic components of depression. Each question has four responses ranging from 0 to 3 points, with higher scores indicating higher levels of depression. It is the gold standard for screening for depression in individuals aged 13 years and older. The maximum total score for all 21 items is 63. BDI-II scores are categorized as minimal or no depression (0–13 points), mild depression (14–19 points), moderate depression (20–28 points), or severe depression (29–63 points). In this study, we used dichotomous variables, such as no depression (0–13 points) and mild-to-severe depression (14–63 points) [29,30].

The General Anxiety Disorder-7 (GAD-7) scale was used to assess the severity of anxiety. This questionnaire contains 7 items that describe the main symptoms of anxiety experienced over the last two weeks. Each item is scored on a four-point Likert scale. The maximum total score is 21 points. Scores of 5, 10, and 15 are taken as the cutoff points for mild, moderate and severe anxiety, respectively. In this study, we used dichotomous variables, such as no anxiety (0–4 points) and mild-to-severe anxiety (≥5 points) [31,32].

### 2.5. Covariates

Biological sex at birth and date of birth were self-reported. Age was calculated from the date of birth. Height was measured twice with a stadiometer (SECA Model 213; Vogel & Halke; Hamburg, Germany; precision, 0.1 cm; range, 20–205 cm), and weight was defined as the average of two measurements obtained with an electronic scale (SECA Model 869; Vogel & Halke; Hamburg, Germany; precision ±0.15%; range, 2–250 kg). The mean of the two measurements, weight and height, was used to determine the body mass index (BMI) via the following formula (weight [kg]/height [m^2^]) [21]. The data on parental education and family history of depression and anxiety were self-reported by the student participants [33].

The following behavioral conditions were selected as covariates based on the available literature [34]. The possible presence of addiction associated with substance use was assessed via the Alcohol, Smoking, and Substance Involvement Screening Test (ASSIST) [35]. This questionnaire provides a risk score for tobacco and alcohol use that can be categorized at three levels: low risk (0–3 points for tobacco and 0–10 points for alcohol), moderate risk (4–26 points for tobacco and 11–26 points for alcohol), and high risk (>27 points for both) [36]. The Pittsburgh Sleep Quality Index (PSQI) is a self-rated questionnaire that assesses sleep quality and disturbances over a 1-month period. This tool assesses 7 domains: subjective sleep quality, sleep latency, sleep duration, habitual sleep efficiency, sleep disturbances, sleep medication use, and daytime dysfunction. It consists of 19 questions, each scored on a 0–3 scale, with higher scores indicating poorer sleep quality. The PSQI global score was used both continuously (0–21 points) and categorically (good sleep quality: ≤5 points; poor sleep quality: >5 points). Total physical activity (metabolic equivalent of task, METs/week) was calculated via the International Physical Activity Questionnaire Short Form (IPAQ) [37], and total dietary energy intake (kcal/day) was determined via the Food Frequency Questionnaire with 137 items [38] and Spanish food composition tables [39].

The problematic use of social media was assessed with the short version of the Social Media Addiction Questionnaire (ARS-6), which consists of 6 items and uses a 5-point scale (scored from 1 to 5), with the 14th percentile or lower indicating occasional risk, and a percentile above 14 being indicative of higher risk [40].

### 2.6. Statistical Analysis

Medians and interquartile ranges (IQRs) or frequencies (*n*) and percentages (%) were reported for all the quantitative and qualitative data, respectively. The Shapiro-Wilk test (*p*-value > 0.05) and density and quantile-quantile plot methods were used to test the normality of the distributions of continuous variables.

To test the relationships between depression and anxiety categories and the total SCOFF score, generalized linear regression models (GLMs) with a negative binomial distribution were constructed. The GLMs were used to estimate the marginal means of the total score of the SCOFF questionnaire (dependent variable) according to the depression and anxiety categories (independent variables), controlling for potential confounders. Finally, the GLMs with a binomial family and a logit link were used to estimate the odds ratio (OR) of the relationship between the depression and anxiety categories according to the DEB categories (cutoff point of ≥2 on the SCOFF questionnaire) and for each SCOFF item (yes vs. no) (dependent variables analyzed separately).

All the results were stratified by sex because of the importance of analyzing all the main variables of our study separately. Age, BMI, parental education, family history of depression, family history of anxiety, risk of tobacco use, risk of alcohol use, total physical activity, total dietary intake, global sleep quality index, and social media addiction were considered covariates in all the models.

All statistical analyses were conducted via SPSS version 29.0 (IBM Corp, Armonk, NY, USA). Significance was set at a *p*-value < 0.05.

## 3. Results

The descriptive characteristics (median [IQR] or *n* [%]) of the study sample by sex are shown in Table 1. Most of the students were female (71.5%), and the median age was 21.0 years (IQR = 3.0). In the females, the frequency of DEBs was 32.7%, and the frequencies of mild-to-severe depression and mild-to-severe anxiety were 42.0% and 77.5%, respectively. Among the males, the proportion of DEBs was 20.2%, and the proportions of mild-to-severe depression and anxiety were 24.0% and 52.7%, respectively.

**Figure 1:**
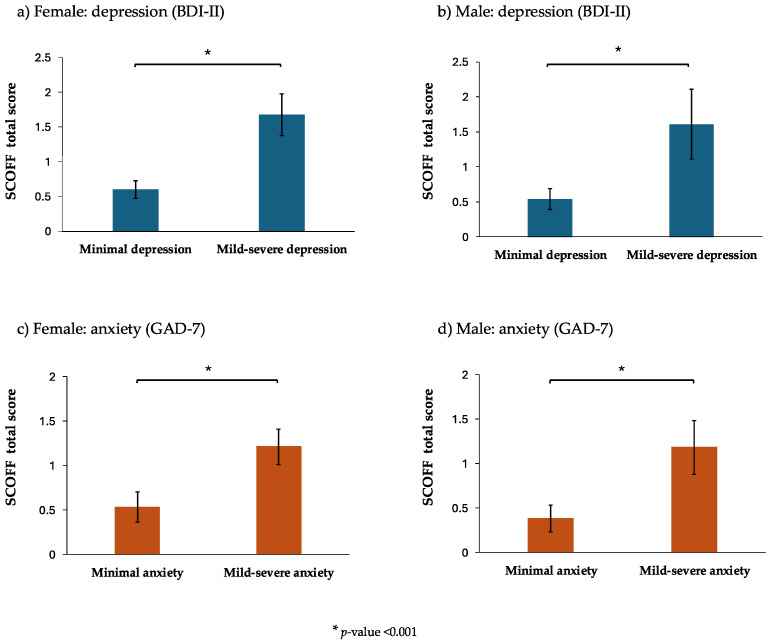
The estimated marginal means of the SCOFF total score (the higher the mean is, the greater the number of disordered eating behaviors, DEBs, there are) in terms of the depression and anxiety categories stratified by sex. The data are shown as columns (estimated marginal means) and vertical lines (95% confidence intervals) obtained with the generalized linear regression models, with a negative binominal distribution adjusted for age, body mass index, parental education, a family history of depression, a family history of anxiety, the risk of tobacco use, the risk of alcohol use, total physical activity, total dietary intake, the global sleep quality index, and social media addiction. Abbreviations: BDI-II: Beck Depression Inventory, second version; DEBs: disordered eating behaviors; GAD-7: Generalized Anxiety Disorder 7-items; SCOFF: Sick, Control, One stone, Fat, Food Questionnaire. * *p*-value < 0.001.

Figure 1 shows the estimated marginal means of DEBs (SCOFF total score) by depression and anxiety categories in both sexes. The females and the males with mild-to-severe levels of depression and anxiety presented significantly higher values of DEBs (SCOFF total score) than their peers classified as having the lowest levels of depression and anxiety after adjusting for potential confounders. The full results of the GLM assessing the relationships between the independent variables (depression and anxiety categories) and the dependent variable (SCOFF total score) can be found in Tables S1 and S2 (Supplementary Materials).

Table 2 presents the adjusted odds ratios of DEBs (SCOFF ≥ 2 points) and the analysis of the five disordered eating behavior symptoms, according to the different items of the SCOFF, in the females and the males. The adjusted models revealed that in the females, the likelihood of DEBs was greater in those with depression (OR = 4.88, 95% CI: 2.65, 8.97) and those with anxiety (OR = 4.14, 95% CI: 1.77, 9.77). In the males, the results from the adjusted models revealed that depression (OR = 2.67, 95% CI: 0.75, 9.20) was not associated with DEBs after the covariates were included, whereas anxiety (OR = 5.68, 95% CI: 1.33, 24.15) was associated with this condition.

Among the females, depression was associated with an increased likelihood of a positive response to items 1, 2, 4, and 5 of the SCOFF questionnaire (i.e., self-induced vomiting, the loss of control over overeating, body image distortion, and the impact of food on life), whereas anxiety increased the risk for items 1, 4, and 5. Among the males, however, both depression and anxiety were associated with an increased risk of a positive response to only item 2 (i.e., the loss of control over overeating).

## 4. Discussion

The results obtained in this study indicate that higher levels of depression and anxiety are associated with a greater likelihood of exhibiting DEBs, which is in line with previous research demonstrating the close relationship between psychological distress and the emergence of dysfunctional eating patterns. Additionally, our findings suggest that these associations are comparable among both females and males, although the DEB frequencies, as well as depression and anxiety, are greater in females.

Mental health problems are often closely interconnected [41]. Depression and anxiety often co-occur because they share biological, psychological, and environmental risk factors that also contribute to the development of DEBs [42,43,44,45,46,47]. These differences may be explained by physiological factors—such as hormonal variations—and cultural factors, in which traditional gender roles influence how emotions are perceived and managed, as well as how individuals respond to societal expectations [42].

The transition to university represents a critical period of vulnerability for students, characterized by major life changes and heightened academic and social expectations. These challenges, combined with newfound independence and the pressure to adapt to complex social dynamics, may contribute to increased levels of stress, anxiety, and depression [48]. In addition to this context, the societal and media pressures surrounding body image further exacerbate the situation, often leading many students to adopt disordered eating behaviors to meet unrealistic aesthetic standards [49], thereby facilitating the development of EDs [50,51]. This phenomenon not only affects their physical and mental health, but can also negatively impact their academic performance [52].

Historically, EDs have been more extensively studied in women because of their higher prevalence in this group [53]; however, it is now widely acknowledged that EDs are not exclusive to females [54]. Generally, women tend to pursue weight loss due to a distorted perception of ideal body size, which is influenced by media- and society-promoted ideals of thinness [55]. Conversely, men are often pressured to attain an ideal body characterized by a muscular “V-shaped” physique, which can lead to dissatisfaction with their musculature and the desire to modify their weight to improve body composition [50,56]. These gendered differences contribute to the tendency for women to engage in risk behaviors such as induced vomiting or strict dieting, whereas men may be more likely to resort to practices such as steroid or hormone use [57].

The traditional conceptions of the ideal body make it more difficult to detect EDs in men, as their symptoms do not always align with the conventional understanding of ED manifestations. This may result in the under-recognition and reduced visibility of ED cases in the male population [41]. In recent years, an increase in the prevalence of EDs has been reported, potentially linked to the changes introduced by the Diagnostic and Statistical Manual of Mental Disorders, Fifth Edition (DSM-5) diagnostic criteria, which improve detection [58]. This evolution underscores the importance of using effective tools to capture the full spectrum of ED symptoms, with the SCOFF questionnaire being among the most recommended instruments for early ED detection [58].

This is the first study in which each item of the SCOFF scale has been individually analyzed and disaggregated by sex. Among the women, the most endorsed items were related to behaviors such as self-induced vomiting, body image distortion, the loss of control over overeating, and the perception that food dominated their lives. In contrast, among the men, the predominant items were associated primarily with the loss of control over food intake. These differences suggest sex-specific patterns in the manifestation of EDs, which may be relevant for both diagnosis and treatment. However, a significant proportion of university students of both sexes expressed concerns about their body image, which may indicate the possible presence of a subclinical ED. Behaviors such as the loss of control over food intake or perceiving oneself as fat despite being thin are common and may indicate atypical eating behaviors. This finding suggests that even a single positive response on the SCOFF questionnaire should be carefully monitored, as it may reflect the early risk of developing an ED [59].

This study has several limitations that should be acknowledged. First, the cross-sectional nature of our analysis prevents us from establishing definitive causal relationships. Second, our findings cannot be generalized to the general young adult population because our analysis focused exclusively on healthy adults enrolled in university studies. Another limitation is that all the data were obtained through self-report questionnaires, which may have introduced recall and social desirability biases in the responses. Finally, the large confidence intervals for some of our estimates may indicate greater uncertainty in the results and reduce the precision and reliability of our conclusions. This was particularly evident in the male population in our sample. This may have affected our ability to detect associations between anxiety or depressive symptoms and certain SCOFF items. Further studies with larger sample sizes for each sex are recommended to either confirm or refute our findings. On the other hand, as strengths of this study, it is worth highlighting the use of validated tools, statistical analysis adjusted for numerous variables, and stratification by sex.

## 5. Conclusions

This study showed a cross-sectional association between higher levels of depression and anxiety symptoms and an increased likelihood of reporting DEBs among Spanish university students. While the strength of the associations between depression, anxiety, and DEBs did not differ significantly by sex, the females reported worse psychological and DEB symptoms than the males. This suggests that while the mechanisms linking mental health and DEBs may be similar for both sexes, the prevalence and specific manifestations of these conditions differ. In this sense, these findings underscore the need for targeted mental health strategies in university settings that consider sex differences. Specifically, universities should implement prevention and intervention programs that include routine mental health screenings, psychoeducational workshops on emotional regulation and body image, and accessible psychological support services. These programs should be tailored to address the greater vulnerability among female students, while ensuring that male students also receive adequate support and awareness, fostering a more inclusive and responsive mental health framework.

## Figures and Tables

**Table 1:** Sociodemographic and lifestyle characteristics of Spanish university students globally and stratified by sex.

Characteristic	Total (*n* = 453)	Female (*n* = 324)	Male (*n* = 129)
Age (years)	21.0 (3.0)	20.0 (3.0)	21.0 (3.0)
BMI (kg/m^2^)	22.4 (5.2)	22.1 (5.2)	23.5 (4.5)
One or more parents with university degree	153 (33.8)	97 (29.9)	56 (43.4)
Family history of depression	35 (7.7)	29 (9.0)	6 (4.7)
Family history of anxiety	31 (6.8)	27 (8.3)	4 (3.1)
Risk of tobacco use	167 (34.9)	123 (37.8)	45 (34.4)
Risk of alcohol use	77 (16.9)	49 (15.1)	28 (21.3)
Sleep quality (PSQI global score) #	6.0 (5.0)	6.0 (4.0)	5.0 (4.0)
Total physical activity (METs/week)	2772.0 (3566.2)	2079.0 (2926.8)	4159.5 (3989.2)
Total dietary energy intake (kcal/day)	2491.3 (1275.7)	2446.7 (1117.0)	2581.2 (1684.7)
Risk of problematic use of social media			
Occasional risk	69 (15.2)	40 (12.3)	29 (22.5)
More than occasional	384 (84.8)	228 (70.4)	87 (67.4)
Disordered eating behavior *			
No	321 (70.9)	218 (67.3)	103 (79.8)
Yes	132 (29.1)	106 (32.7)	26 (20.2)
SCOFF total score	1.0 (2.0)	1.0 (2.0)	0.0 (1.0)
Depression			
No depression	286 (63.1)	188 (58.0)	98 (76.0)
Mild-to-severe depression	167 (36.9)	136 (42.0)	31 (24.0)
Mild depression	80 (17.7)	60 (18.5)	20 (15.5)
Moderate depression	54 (11.9)	47 (14.5)	7 (5.4)
Severe depression	33 (7.3)	29 (9.0)	4 (3.1)
BDI-II total score	10.0 (12.0)	11.0 (13.0)	8.0 (9.0)
Anxiety			
No	134 (29.6)	73 (22.5)	61 (47.3)
Mild-to-severe anxiety	319 (70.4)	251 (77.5)	68 (52.7)
Mild anxiety	158 (34.9)	114 (35.2)	44 (34.1)
Moderate anxiety	104 (23.0)	86 (26.5)	18 (14.0)
Severe anxiety	57 (12.6)	51 (15.7)	6 (4.7)
GAD-7 total score	7.0 (7.0)	8.0 (7.0)	5.0 (6.0)

Values are medians and IQRs (for continuous variables) or counts (%) for categorical variables. Abbreviations: BDI-II: Beck Depression Inventory, second version; BMI: body mass index; GAD-7: Generalized Anxiety Disorder 7-items; MET: metabolic equivalent of task; PSQI: Pittsburgh Sleep Quality Index; SCOFF: Sick, Control, One stone, Fat, Food Questionnaire. # A higher score indicates poorer sleep quality. * Cutoff points for disordered eating behavior ≥ 2 points on the SCOFF questionnaire.

**Table 2:** Odds ratios (95% confidence intervals) for associations between depression and anxiety with disordered eating behaviors and each item of SCOFF questionnaire stratified by sex.

Exposure	Cases/ Total	DEBs (SCOFF Total Score ≥ 2)	Item 1 (Yes vs. No)	Item 2 (Yes vs. No)	Item 3 (Yes vs. No)	Item 4 (Yes vs. No)	Item 5 (yes vs. no)
**Female**							
** Depression (BDI-II)**							
Crude model							
No depression	188/324	Ref.	Ref.	Ref.	Ref.	Ref.	Ref.
Mild-to-severe depression	136/324	6.23 (3.73, 10.39) *	5.51 (2.91, 10.44) *	3.18 (2.01, 5.05) *	1.77 (0.84, 3.73)	6.22 (3.39, 11.42) *	6.01 (2.93, 12.31) *
Adjusted model							
No depression	188/324	Ref.	Ref.	Ref.	Ref.	Ref.	Ref.
Mild-to-severe depression	136/324	4.88 (2.65, 8.97) *	3.99 (1.95, 8.20) *	2.26 (1.30, 3.91) *	1.20 (0.49, 2.98)	4.54 (2.23, 9.24) *	5.33 (2.35, 12.10) *
** Anxiety (GAD-7)**							
Crude model							
No anxiety	73/324	Ref.	Ref.	Ref.	Ref.	Ref.	Ref.
Mild-to-severe anxiety	251/324	5.20 (2.39, 11.31) *	6.03 (2.85, 12.77) *	1.64 (0.95, 2.84)	2.07 (0.70, 6.14)	4.65 (1.79, 12.05) *	4.33 (2.02, 9.28) *
Adjusted model							
No anxiety	73/324	Ref.	Ref.	Ref.	Ref.	Ref.	Ref.
Mild-to-severe anxiety	251/324	4.14 (1.77, 9.77) *	4.63 (2.09, 10.50) *	1.18 (0.63, 2.19)	1.53 (0.47, 4.97)	3.01 (1.09, 8.33) *	3.93 (1.59, 9.69) *
**Male**							
** Depression (BDI-II)**							
Crude model							
No depression	98/129	Ref.	Ref.	Ref.	Ref.	Ref.	Ref.
Mild-to-severe depression	31/129	7.41 (2.88, 19.04) *	2.75 (0.69, 10.98)	6.28 (2.61,15.07) *	2.30 (0.80, 6.59)	4.84 (1.80, 12.95) *	6.47 (1.93, 21.63) *
Adjusted model							
No depression	98/129	Ref.	Ref.	Ref.	Ref.	Ref.	Ref.
Mild-to-severe depression	31/129	2.67 (0.75, 9.20)	0.62 (0.07, 4.94)	3.36 (1.16, 9.74) *	1.33 (0.36, 4.87)	2.58 (0.71, 9.38)	3.95 (0.77, 12.13)
** Anxiety (GAD-7)**							
Crude model							
No anxiety	61/129	Ref.	Ref.	Ref.	Ref.	Ref.	Ref.
Mild-to-severe anxiety	68/129	6.81 (2.19, 21.18) *	3.29 (2.85, 13.02)	4.53 (1.98, 10.38) *	1.96 (0.68, 5.60)	2.00 (0.75, 5.34)	4.58 (1.39, 15.10) *
Adjusted model							
No anxiety	61/129	Ref.	Ref.	Ref.	Ref.	Ref.	Ref.
Mild-to-severe anxiety	68/129	5.68 (1.33, 24.15) *	2.06 (0.19, 22.13)	4.20 (1.57, 11.20) *	1.32 (0.42, 4.15)	1.72 (0.54, 5.50)	1.65 (0.31, 8.64)

The values expressed as odds ratios (95% confidence intervals) were obtained from the generalized linear regression models with a binomial family and a logit link. In addition to the crude model, the adjusted model controlled for age, body mass index, parental education, a family history of depression, a family history of anxiety, the risk of tobacco use, the risk of alcohol use, total physical activity, total dietary intake, the global sleep quality index, and social media addiction. Item 1, vomiting; item 2, the loss of control overeating; item 3, dieting for weight loss; item 4, body image distortion; and item 5, the impact of food on life. BDI-II: Beck Depression Inventory, second version; DEBs: disordered eating behaviors; GAD-7: Generalized Anxiety Disorder 7-items; SCOFF: Sick, Control, One stone, Fat, Food Questionnaire. * *p*-value < 0.001.

## Data Availability

Data will be available upon reasonable request to the corresponding author.

## Supplementary Materials

The following supporting information can be downloaded at https://www.mdpi.com/article/10.3390/jcm14134611/s1, Table S1. Association between depression categories and Sick, Control, One stone, Fat, Food (SCOFF) total score by sex; Table S2. Association between anxiety categories and Sick, Control, One stone, Fat, Food (SCOFF) total score by sex.
