# Supplementary material for: Cross-Sectional Associations Between Depressive and Anxiety Symptoms and Disordered Eating Behaviors by Sex in University Students
Source: J Clin Med. 2025 Jun 29;14(13):4611. doi: 10.3390/jcm14134611 (PMC12250445; doi:10.3390/jcm14134611)
Supplement: Supplementary file 1 [file jcm-14-04611-s001.zip › jcm-3700817-supplementary.pdf]

Supplementary Materials

Table S1. Association between depression categories and Sick, Control, One, Fat and Food (SCOFF) total score by sex.

Predictor	*β*	SE	LLCI	ULCI	p-value
Female					
Minimal depression	Reference				
Mild-severe depression	1.03	0.16	0.71	1.35	<0.001
Male					
Minimal depression	Reference				
Mild-severe depression	1.09	0.28	0.53	1.65	<0.001

Abbreviations: B, unstandardized beta coefficient; LLCI, lower limit confidence interval; SE, standard error; ULCI, upper limit confidence interval.

Table S2. Association between anxiety categories and Sick, Control, One, Fat and Food (SCOFF) total score by sex.

Predictor	*β*	SE	LLCI	ULCI	p-value
Female					
Minimal anxiety	Reference				
Mild-severe anxiety	0.82	0.22	0.39	1.24	<0.001
Male					
Minimal anxiety	Reference				
Mild-severe anxiety	1.14	0.29	0.56	1.71	<0.001

Abbreviations: B, unstandardized beta coefficient; LLCI, lower limit confidence interval; SE, standard error; ULCI, upper limit confidence interval.
